# Supplementary material for: Deletion of wfs1 Impairs Oligodendrocyte Precursor Cells Dorsal Distribution and Myelination Through the wfs1-hmgcs1 Axis in Zebrafish
Source: Biology (Basel). 2026 Aug 16;15(16):1402. doi: 10.3390/biology15161402 (PMC13510151; doi:10.3390/biology15161402)
Supplement: Supplementary file 1 [file biology-15-01402-s001.zip › File S1-Transcriptome sequencing data and differentially expressed genes (DEGs) in wfs1-- zebrafish at 5 dpf/1.GO/wfs1KOvsWT/ALL/wfs1KOvsWT.all_GObar_cc.pdf]

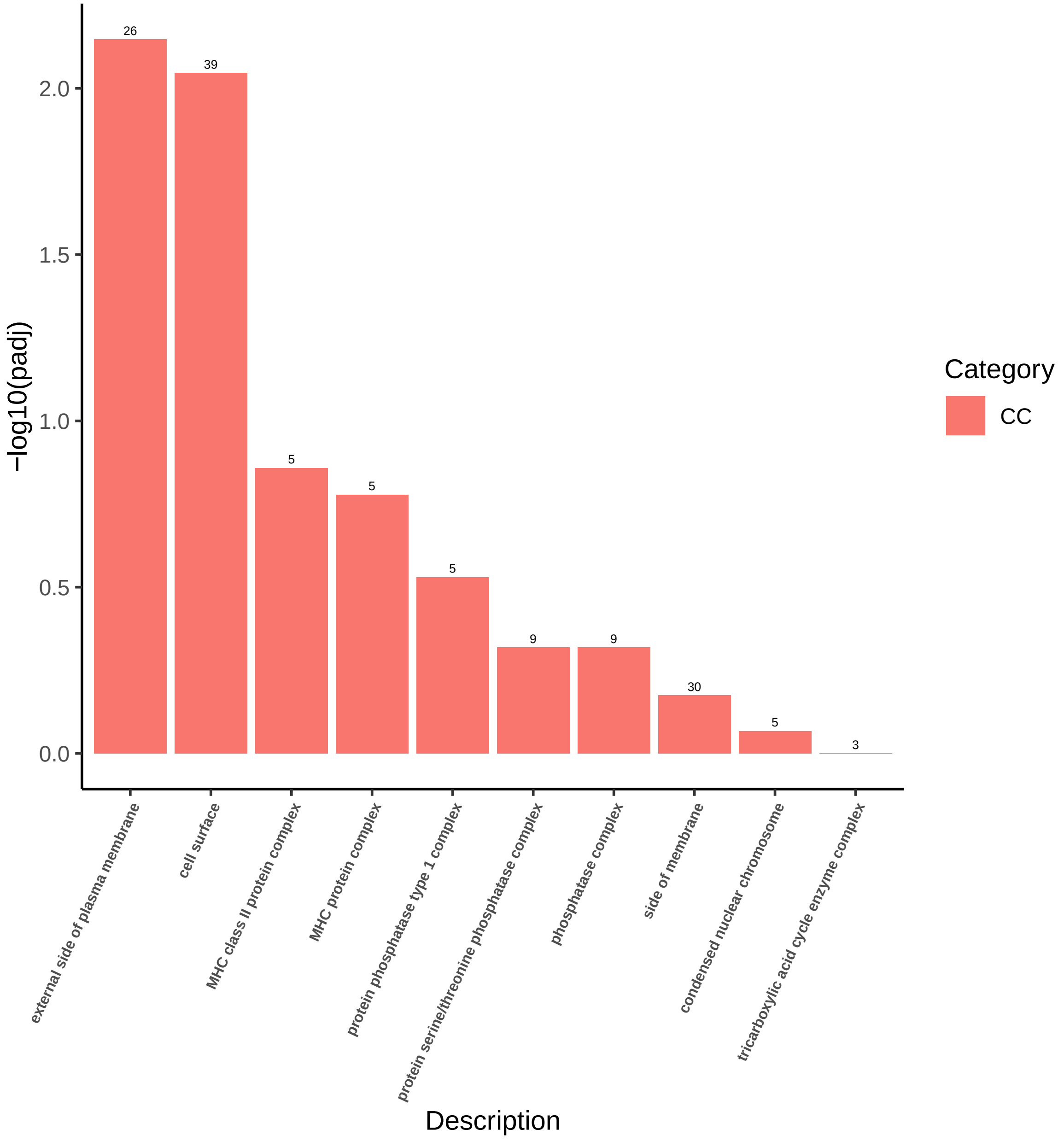

−log10(padj)
2.0
1.5
1.0
0.5
0.0
26
39
5
5
5
9
9
30
5
3
external side of plasma membrane
cell surface
MHC class II protein complex
MHC protein complex
protein phosphatase type 1 complex
protein serine/threonine phosphatase complex
phosphatase complex
side of membrane
condensed nuclear chromosome
tricarboxylic acid cycle enzyme complex
Description
Category
CC
